# Online Multiple Athlete Tracking with Pose-Based Long-Term Temporal Dependencies

**DOI:** 10.3390/s21010197

**Published:** 2020-12-30

**Authors:** Longteng Kong, Mengxiao Zhu, Nan Ran, Qingjie Liu, Rui He

**Affiliations:** 1 School of Computer Science and Engineering, Beihang University, Beijing 100191, China; konglongteng@buaa.edu.cn (L.K.); nknanran@buaa.edu.cn (N.R.); heruihr@buaa.edu.cn (R.H.); 2 State Key Laboratory of Software Development Environment, Beihang University, Beijing 100191, China; zhumx@buaa.edu.cn; 3 Hangzhou Innovation Institute, Beihang University, Hangzhou 310000, China

**Keywords:** sports video analysis, Multi-Athlete Tracking (MAT), long short-term memory (LSTM) networks

## Abstract

This paper addresses the Multi-Athlete Tracking (MAT) problem, which plays a crucial role in sports video analysis. There exist specific challenges in MAT, e.g., athletes share a high similarity in appearance and frequently occlude with each other, making existing approaches not applicable for this task. To address this problem, we propose a novel online multiple athlete tracking approach which make use of long-term temporal pose dynamics for better distinguishing different athletes. Firstly, we design a Pose-based Triple Stream Network (PTSN) based on Long Short-Term Memory (LSTM) networks, capable of modeling long-term temporal pose dynamics of athletes, including pose-based appearance, motion and athletes’ interaction clues. Secondly, we propose a multi-state online matching algorithm based on bipartite graph matching and similarity scores produced by PTSN. It is robust to noisy detections and occlusions due to the reliable transitions of multiple detection states. We evaluate our method on the APIDIS, NCAA Basketball and VolleyTrack databases, and the experiment results demonstrate its effectiveness.

## 1. Introduction

In recent years, sports video analysis has received increasing attention in academia and industry due to its scientific challenges and promising applications. It covers various application scenarios or research directions, including automatic game commentary, tactical analysis, player statistics, etc. Among these directions, athletes tracking is fundamental and critical for sports video analysis.

In the literature, several early attempts [1,2,3] focus on Multi-Athlete Tracking (MAT) in volleyball, basketball and soccer game videos. They generally apply additional pre-processing to simplify tracking based on additional clues within the sports fields, e.g., site boundaries and static cameras. Xing et al. [3] build an observation model to classify the playfield region and nonplayfield regions with color information, providing convenience for athlete localization. Gomez et al. [1] make use of static cameras for separating foreground and background easily, and thus producing clean athlete targets for tracking. However, those methods are probably not suitable for complex scenes in the wild, which contain variations in background and illumination.

Actually, most efforts are made to track multiple athletes [4,5] following the typical Multi-Object Tracking (MOT) framework, which employs the tracking-by-detection paradigm, i.e., associating the prepared detections in terms of different objects at each frame. The MOT methods can be roughly categorized into two branches, i.e., online matching based and offline association based, according to whether using subsequent frames. Offline association approaches often show better tracking performance as additional information is employed; however, they always require higher computational cost. While the online matching ones have advantages in high processing speed and applicability. As in the popular benchmarks, e.g., PETS, UA-DETRAC, and MOTChallenge, they pay more attention to pedestrians and vehicles in surveillance scenes, which have stable appearances and motions.

Indeed, in real sports scenes, there exist some specific difficulties: (1) athletes share a high similarity in appearance (dressing, figure, etc), and they frequently occlude with each other; (2) athletes often have abrupt positions and complex actions. These facts make the existing MOT methods [6,7,8,9], especially the ones focus on appearance and simple motion clues, lose efficacy. When playing games, the athletes usually have their own specific pose dynamics, which are distinct from each other within a period of time. For example, when the setter is passing the ball, the spiker is waiting for attacking. This brings us the idea that long-term temporal pose dynamics may help to distinguish different athletes. Based on the considerations above, we propose a novel multiple athlete tracking approach to the given issue, following the popular online tracking-by-detection paradigm.

Specifically, we first design a Pose-based Triple Stream Network (PTSN) based on Long Short-Term Memory (LSTM) networks, capable of modeling long-term temporal pose dynamics of athletes. Moreover, to capture more subtle differences between athletes, we enrich the pose dynamic into three clues, i.e., pose-based appearance, motion and interactions of athletes, which are modeled by three network streams in PTSN. Given the history tracklet and current detection, PTSN could generate robust affinity between them according to the degree of dependency. Second, we design a multi-state online matching algorithm based on bipartite graph matching. It uses the affinities produced by PTSN to associate the athlete detections frame by frame and finally accomplish the tracking. More importantly, in the online association, we define multiple detection states and build reliable transitions, boosting the association robustness to noisy detections and occlusion.

In summary, the main contributions of this paper are highlighted as follows:

- We propose a Pose-based Triple Stream Networks (PTSN) based on Long Short-Term Memory (LSTM) networks, capable of modeling long-term temporal pose dynamics of athletes and generating robust association affinities.
- We design a multi-state online matching algorithm based on multiple detection states and reliable transitions with the association affinities, improves the robustness to noisy detections and occlusion.
- We evaluate our method by comparing it with recently proposed advanced multi-object trackers on the APIDIS, NCAA Basketball and VolleyTrack databases, and the experiment results demonstrate the effectiveness of our method.

A preliminary version of this work is presented in [10]. Compared with the conference version, the extensions include: (1) more details of the proposed approach and related works are explained; (2) more experiments on APIDIS and NCAA databases are conducted and more results are displayed and discussed.

The rest of this paper is organized as follows. In Section 2, related works for athlete tracking and multiple object tracking are discussed. Section 3 describes our MAT approach in detail, including the Pose-based Triple Stream Network and multi-state online matching algorithm. In Section 4, databases, experimental results and analysis are shown. Finally, Section 5 draw an conclusion for the paper with perspectives.

## 2. Related Works

### 2.1. Athlete Tracking

In the past few decades, despite a few works focusing on single athlete tracking [11], most studies address the Multi-Athlete Tracking problem. Some early attempts make use of specific clues in sports fields (e.g., site boundaries) to facilitate athlete tracking. Xing et al. [3] introduce a progressive observation modeling strategy to classify the playfield region and nonplayfield regions with color information. Based on classification results, the tracking component is achieved by a Bayesian inference approach. Mauthner [2] and Gomez [1] use particle filters to predict positions and velocities of players in beach volleyball games. They separate foreground and background to make athlete modeling easier, but cues in background may lose potentially useful in improving the stability and accuracy of trackers. However, those methods may lack practicability in more complex scenes due to the variations in background and illumination. Meanwhile, more studies take MAT as an MOT problem and process it in the tracking-by-detection framework. Liu et al. [4] tracked players in basketball and hockey game videos from the view of tactics analysis. They try to predict all the possible moving directions of players, but it may incur failure due to infinite possibilities. Shitrit et al. [5] propose a Multi-Commodity Network Flow approach to track multiple players in basketball and soccer games. They exploit both the appearance and position clues to prevent identity switches, and report promising results.

### 2.2. Multi-Object Tracking

There exist a number of approaches attempting to address the MOT problem, and they can be roughly divided into traditional ones and deep learning based ones. Further, traditional methods can be classified into online matching based [12,13,14,15] and offline association based [16,17,18,19,20,21]. The offline association approaches take advantages of global information and prove more robust to complex scenes. They cast the task of MOT as a graphical model solving problem, where the graphs are built with a set of detections or tracklets. Many globally optimal techniques, e.g., k-shortest paths in DP_NMS [18], Linear Program in ELP [20], are used to solve the graphical models. Recently, more robust pairwise affinities based on strong appearance cues, e.g., sparse appearance models and integral channel feature appearance, are proved effective in associations. This lead the online matching methods, e.g., MHT_DAM [9] and LINF1 [22], achieving state-of-the-art performance.

More recently, Yu et al. [23] took advantage of high-performance detection and representative feature of CNNs, and achieved significantly better results on MOTChallenge [24,25] in both online and offline mode; Leal-Taixé et al. [26] defined a new CNN-based structure for people appearance representation to build effective relations between detections. Sadeghian et al. [6] presented a structure Recurrent Neural Networks (RNNs) based network architecture that reasons jointly on multiple cues over a temporal window. The deep models used in those methods largely enhance the tracklet affinity and improve the performance for both online matching and offline association. However, most existing MOT methods pay more attention to the pedestrian tracking in surveillance, short of investigation on multiple athlete tracking in sports videos.

### 2.3. Human Pose Estimation

In the literature, many studies [27,28,29] explore the pose clue for person tracking for its rich semantic information, and they take full advantage of human pose estimation (HPE) task [30,31,32,33]. Recent human pose estimation methods can be classified into bottom-up and top-down ones. Bottom-up approaches [34,35] first detect individual body joints and then group them into people, while the top-down ones [32,33] first detect people bounding boxes and then predict their joint locations within each region. Top-down approaches do not need any joint grouping due to the detectors. Correspondingly, bottom-up approaches are lack of this reliance on a detector. Following the Tracking-By-Detection (TBD) paradigm, some methods [28,29] use the top-down technique to produce the human pose. However, they only use the joints feature, which does not generalize well for representing persons. In our work, we enrich the pose dynamic into multiple pose-based clues, which improves the representation ability.

## 3. Multi-Athlete Tracking Approach

As mentioned in Section 1, MAT can be treated as a special case of MOT, which aims at estimating the successive states (e.g., position and size or bounding box) of concerned objects (e.g., pedestrians and vehicles) in the video frames. Correspondingly, we follow the commonly used Tracking-By-Detection (TBD) paradigm to solve the MAT problem, i.e., given athlete detections (producing by Faster R-CNN [36] detector) at each frame, we aim at matching their identities across different frames to generate a set of athletes trajectories over time.

**Figure 1:**
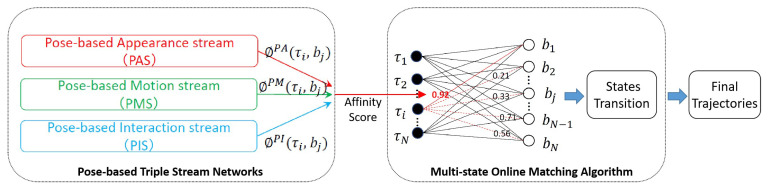
The proposed Multi-Athlete Tracking Framework.

Our framework, as shown in Figure 1, consists of the Pose-based Triple Stream Networks (PTSN) and a multi-state online matching algorithm. PTSN is responsible for calculating the similarity scores between the history tracklets and the candidate detection in the current frame, where the scores come from three network streams that model three pose clues, i.e., pose-based appearance, motions and athletes’ interactions. Based on the similarity scores, the multi-state online matching algorithm generates final trajectories for athletes by tracklet/detection matching and state transitions. Details of our proposed PTSN and the matching algorithm are described in the following.

### 3.1. Overall Architecture of PTSN

The overall architecture of PTSN is shown in left-side of Figure 1. It is comprised of three streams, including Pose-based Appearance Stream (PAS), Pose-based Motion Stream (PMS) and Posed-based Interaction Stream (PIS). They generate similarity scores $\phi^{PA}\left(\tau_{i},b_{j}\right)$, $\phi^{PM}\left(\tau_{i},b_{j}\right)$ and $\phi^{PI}\left(\tau_{i},b_{j}\right)$ respectively, which are further fused into the final one $\phi (\tau_{i},b_{j})$ for connecting the history tracklet $\tau_{i}$ and the current detection $b_{j}$. The details of the three streams of PTSN will be explained in the following three subsections.

To encode the long-term dependencies of sequences, we use LSTM as the main structure in our networks. Unlike popular graph-based tracking methods [14,16,37], whose similarity scores are only calculated in the previous frame, our method could capture the long-term dependencies of targets by inferring from the observation sequences of variable length.

### 3.2. Pose-Based Appearance Stream(PAS)

**Figure 2:**
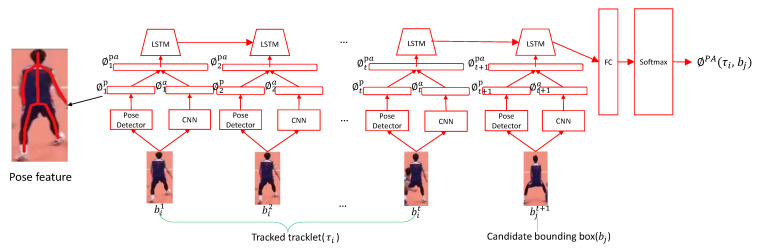
The architecture of Pose-based Appearance Stream (PAS). The inputs are $\tau_{i}$ and $b_{j}$. $\tau_{i}$ is tracklet of *i*th athlete, composed of his bounding boxes from time 1 to t, and $b_{j}$ is a candidate detection at time t+1. The concatenated features (i.e., pose features and appearance features) are fed into an LSTM followed by a softmax layer to generate the similarity score $\phi^{PA}\left(\tau_{i},b_{j}\right)$.

As we introduced above, the athletes share very similar appearances when playing games. To improve the discrimination among athletes, besides the appearance cues, we employ the pose and position information. The Pose-based Appearance Stream (PAS) is then designed, as shown in Figure 2, which models the long-term evolution of pose and appearance. It takes a tracklet and a detection as input and estimates their affinity, i.e., determining whether the candidate detection box contains the same athlete in the tracklet. A tracklet is a set of bounding boxes of tracked athlete’s trajectory at timesteps 1,...,t, i.e., $\tau_{i}=\left(b_{i}^{1},b_{i}^{2},...,b_{i}^{t}\right)$, and the candidate bounding box $b_{j}^{t+1}$ is the detection in time t+1.

Specifically, we concatenate the tracklet and detection to construct a new detection sequence, extract the pose-based appearance features that are fed into the LSTM network to model the long-term evolutions. Then we use Softmax followed by the LSTM for a binary classification problem, estimating how confident the evolutions come from the same athlete.

At each frame, the pose and appearance feature of the detected athlete are extracted by the feature extractor, where the pose coordinates are mapped into original frames, therefore including the athlete positions. We use the output of the last layer of Hourglass Networks [32] as pose feature $\phi^{p}$. For the athlete in a bounding box, we use the pretrained model of the Hourglass Networks on COCO dataset to generate *x* and *y* coordinates of 16 pose joints, leading to a pose feature of *H*-dimension. We start with the initial weights of ResNet-101 pre-trained on ImageNet and add a new fully connected layer on the top of the structure to extract a *H*-dimensional appearance feature $\phi^{a}$. Given the detection sequence, the extracted pose feature sequence, i.e., $\left(\phi_{1}^{p},\phi_{2}^{p},...,\phi_{t}^{p},\phi_{t+1}^{p}\right)$, and appearance feature sequence, i.e., $\left(\phi_{1}^{pa},\phi_{2}^{pa},...,\phi_{t}^{pa},\phi_{t+1}^{pa}\right)$ are combined, forming the input feature sequence of LSTM, i.e., $\left(\phi_{1}^{pa},\phi_{2}^{pa},...,\phi_{t}^{pa},\phi_{t+1}^{pa}\right)$, where $\phi_{t}^{pa}$ is a 2H-dimensional feature vectors at *t* frame, $\phi_{t+1}^{pa}$ is the feature of candidate detection. The LSTM takes the feature sequence as input, and models the long-term dependencies of pose-based appearance, which is carried by a hidden representation $h_{pa}$. $h_{pa}$ is then fed into a softmax classification layer (contains a fully-connected layer and a softmax layer) to output a score $\phi^{PA}\left(\tau_{i},b_{j}\right)$, i.e., the tracklet-detection affinity.

### 3.3. Pose-Based Motion Stream (PMS)

**Figure 3:**
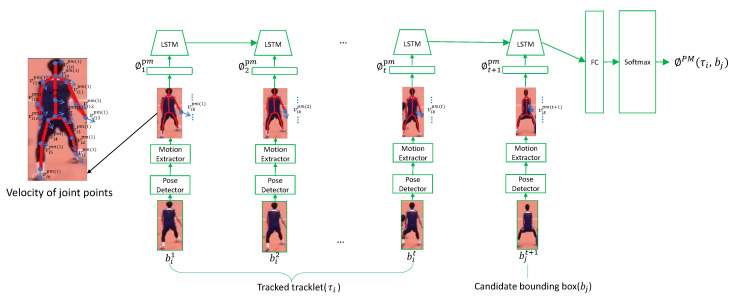
The architecture of Pose-based Motion Stream (PMS). The inputs are τ and $b_{j}$. τ is tracklet of *i*th athlete, composed of his bounding boxes from time 1 to t, and $b_{j}$ is a candidate detection at time t + 1. The motion features are fed into an Long Short-Term Memory (LSTM) followed by a softmax layer to generate the similarity score $\phi^{PM}\left(\tau_{i},b_{j}\right)$. Velocity definition of body joints can be seen in left-side.

The motion information is another critical clue for distinguishing athletes, for instance, the velocity variation of the athletes usually differ significantly. We design the Pose-based Motion Stream (PMS), illustrated in Figure 3, which can estimate tracklet-detection affinity based on athlete motion dynamics. Instead of representing motion by using the velocity of the center of an athlete’s bounding box, PMS computes the velocity of each joint to describe motion information, delivering a more comprehensive representation of the athlete.

PMS consists of a pose detector, a motion detector, and an LSTM network followed by a softmax classification layer. The pose detector is the same as the one in PAS. For each image region in the tracklet, the 16 pose joints, i.e., *x*, *y* coordinates in original image, of an athlete are extracted. Based on the pose coordinates, we first calculate the joint velocities and transform them into embedding space. Assume that $V_{ik}^{pm(t)}$ refer to the *k*th joint velocity of the *i*th athlete at *t*th frame, it can be calculated as follows:

(1) $$V_{ik}^{pm(t)}=\left(V_{ik(x)}^{pm(t)},V_{ik(y)}^{pm(t)}\right)=\left(X_{ik}^{pm(t-1)}-X_{ik}^{pm(t)},Y_{ik}^{pm(t-1)}-Y_{ik}^{pm(t)}\right)$$

where $\left(X_{ik}^{pm(t)},Y_{ik}^{pm(t)}\right)$ are the 2D coordinates of *i*th athlete on the *k*th joint at *t*th frame. To improve the abstract ability of PAS, we transform the joint velocities $V_{ik}^{pm(t)}$ into embedding space of higher dimensions by a fully-connected layer. Given the input tracklet $\tau_{i}=\left(b_{i}^{1},b_{i}^{2},...,b_{i}^{t},b_{j}^{t+1}\right)$, $b_{j}^{t+1}$ is the candidate detection, after post/motion extraction, we can obtain the velocity feature sequence $\phi^{pm}=\left(\phi_{1}^{pm},\phi_{2}^{pm},...,\phi_{t}^{pm},\phi_{t+1}^{pm}\right)$, where $\phi_{t}^{pm}$ is a *H*-dimensional feature vectors at *t* frame. Then $\phi^{pm}$ passes through an LSTM layer. The hidden state $h_{m}$ of this LSTM layer carries dynamics of pose-based motion information. We take the final time-step of $h_{m}$ as global representation and feed it into a softmax classification layer with cross-entropy loss to predict the tracklet-detection affinity score $\phi^{PM}\left(\tau_{i},b_{j}\right)$.

### 3.4. Pose-Based Interaction Stream (PIS)

**Figure 4:**
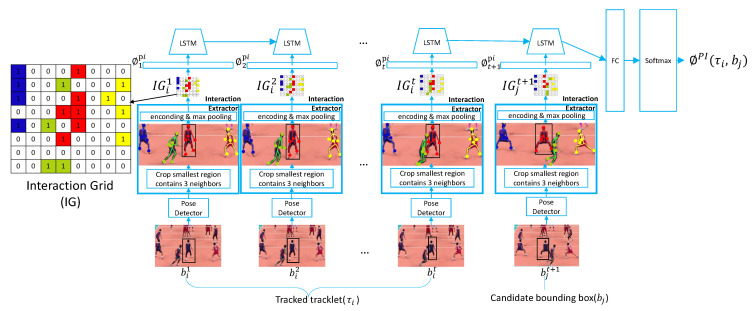
The architecture of Posed-based Interaction Stream (PIS). A pose detector is applied to obtain pose information of an athlete from previous *t* frames. The interaction grids of this athlete are calculated between his/her closest 3 neighbors at each frame. Then we apply an LSTM to encode interaction information for this athlete and compare it with candidate boxes $b_{j}$ generated by the detector at timestep t+1. Finally, the LSTM outputs a similarity score $\phi^{PI}\left(\tau_{i},b_{j}\right)$ indicating the probability of the candidate boxes containing the same athlete.

Both pose-based appearance and motion clues are used to represent individual athletes, however, they lose the contextual structure, which is also an important discriminative information. Therefore, we propose the Pose-based Interaction Stream (PIS), as shown in Figure 4, which can capture the interactions among a specific athlete and the ones around him/her. Since the number of neighbors can vary, in order to obtain the same size input, we model the neighborhood of each target as a fixed size grid and design the Interaction Grid (IG). In IG, each athlete is represented as six main joint positions, including head, left wrist, right wrist, left ankle, right ankle and mean value of all joint positions. Assume that $\left(IG_{i}^{1},IG_{i}^{2},...,IG_{i}^{t}\right)$ is the interaction grid for the *i*th athlete at timesteps 1,...,t. The pose joint positions are pooled into the grid and $IG^{t}$ can be defined as:

(2) $$IG_{i}^{t}\left(m,n\right)=\sum_{j\in N_{i},k\in P_{j}}1_{mn}\left[x_{t}^{jk}-x_{t}^{i},y_{t}^{jk}-y_{t}^{i}\right]$$

where $1_{mn}\left[x,y\right]$ is an indicator function to check if the athlete’s joint at (x,y) is in the (m,n) cell of the grid. $N_{i}$ is the set of neighbors of the athlete *i*, $|N_{i}|=3$. $P_{j}$ is the set of joints of neighbor *j*. After building the interaction grids, we use a fully-connected layer that takes the interaction grids as input and produces *H*-dimensional output for each timestep. Similar to PAS and PMS, we also map the interaction grid of candidate detection *j* at timestep t+1 to *H*-dimensional feature vector using the same measure, forming the interaction feature sequence $\phi^{pi}=\left(\phi_{1}^{pi},\phi_{2}^{pi},...,\phi_{t}^{pi},\phi_{t+1}^{pi}\right)$. The feature sequence is fed into an LSTM layer to model the contextual structure, which is carried in its hidden state $h_{i}$. The final step of $h_{i}$ is passed to a softmax classification layer with cross-entropy loss to predict the tracklet-detection affinity score $\phi^{PI}\left(\tau_{i},b_{j}\right)$.

### 3.5. Multi-State Online Matching Algorithm

Based on the final fusion similarity score between a tracklet and detection, we design a multi-state online matching algorithm, shown in Algorithm 1, for accomplishing the final tracking in an online mode.

**Figure 5:**
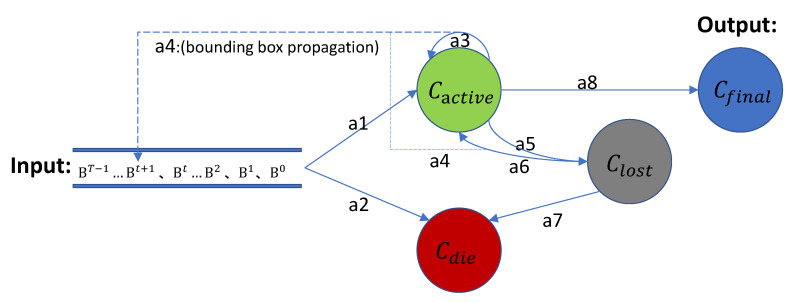
State transition diagram of our matching algorithm, where the input are bounding boxes belong to each frames $\left\{B_{0},B_{1},B_{2},...,B_{T-1}\right\}$. $C_{active}$ is a pooling storing tracklets that have been tracked so far. $C_{lost}$ is a pooling storing tracklets that tracked but lost. $C_{die}$ is a pooling storing tracklets judged to be illegal. $C_{final}$ is a pooling storing legal output tracklets. The transfer actions {*a1, a2, ..., a8*} between them will be explained in detail below, and there will be corresponding annotations in the Algorithm 1.

State transition diagram of our tracker is shown in Figure 5. First of all, a set of bounding boxes belonging to each frames $\left\{B^{0},B^{1},B^{2},...,B^{T-1}\right\}$ is filtered via Non-Maximum Suppression (NMS) operation. High score bounding boxes are selected to next step (as *a1* operation). On the contrary, low score bounding boxes are sent to die tracklets container ($C_{die}$), terminating their life cycles (as *a2* operation). For each high score bounding box $b_{j}$, we feed it into PTSN together with each tracked tracklet $\tau_{i}$ in active tracklets container $C_{active}$ to calculate the tracklet-detection affinity $\sigma_{PTSN}$, where tracked tracklets $C_{active}$ consists of $\left\{\tau_{1},\tau_{2},...,\tau_{i},...,\tau_{n}\right\}$ in previous frames. If the affinity of a detection over $\sigma_{PTSN}$, it will update old $\tau_{i}$ in $C_{active}$ (as *a3* operation) by replacing $b_{j}$ in $\tau_{i}$. Then the bounding box propagation operation will be applied on lost and active containers to predict next bounding box of $\tau_{i}$ in the subsequent frame, according to the velocity of $\tau_{i}$ (as *a4* operation). If the prediction can not catch the next detection, both $b_{j}$ and old $\tau_{i}$ will be sent to next step (as *a5* operation), denoting $\tau_{i}$ as missing tracklet. For the remaining detections, we compare with tracklets in $C_{die}$ for targets recovery. The process will be done for every $b_{j}$. If they match successfully (affinity score over $\sigma_{PTSN}$), they will be sent to $C_{active}$ again (as *a6* operation), and then bounding box propagation operation will be done to predict next bounding box of $\tau_{i}$ in the following frame, according to velocity of $\tau_{i}$(as *a4* operation). If they fail and the waiting time has exceeded the hyper-parameter $\delta_{waiting}$, they will be sent to $C_{die}$, ending its life cycle (as *a7* operation). After that, the remaining bounding box will form a new tracklet and wait for a matching in $C_{lost}$. When the bounding boxes of the last frame $B_{T-1}$ is executed, all tracklet of $C_{active}$ will be copied to $C_{final}$ as output of our tracker as long as they are longer than $\lambda_{min}$ (as *a8* operation). More detailed algorithm steps are strictly illustrated in Algorithm 1.
**Algorithm 1** Multi-State Online Matching Algorithm. **Inputs:**    $B=\left\{B^{0},B^{1},B^{2},...,B^{T-1}\right\}=\left\{{\left\{b_{0},b_{1},...,b_{N-1}\right\}}^{0},...,{\left\{b_{0},b_{1},...,b_{N-1}\right\}}^{T-1}\right\}$ **Outputs:**    $C_{final}$ 1:   Initial: $C_{active}=B^{1}$, $C_{lost}=\phi$, $C_{die}=\phi$, $C_{final}=\phi$ 2:   **for**
t=2 to T−1
**do** 3:    Bt = NMS($B^{t}$) 4:    **for**
$\tau_{i}\in C_{active}$
**do**
 5:     $b_{best}=b_{j}$, where max(PTSN($\tau_{i},b_{j}$)), $b_{j}\in B^{t}$ 6:     **if** PTSN($\tau_{i}$, $b_{j}$) $\geq \sigma_{PTSN}$
**then** 7:       add $b_{best}$ to $\tau_{i}$ and remove $b_{best}$ from $B^{t}$ 8:       predict $b_{p}$ from $\tau_{i}$ and add $b_{p}$ to $B^{t+1}$ 9:     **else** 10:     move $\tau_{i}$ to $C_{lost}$ 11:   **end if** 12:    **end for** 13:    **for**
$\tau_{i}\in C_{lost}$
**do** 14:    $b_{best}$ = $b_{j}$ where max(PTSN($\tau_{i},b_{j}$)), $b_{j}\in B^{t}$ 15:    **if** PTSN($\tau_{i},b_{j}$) $\geq \sigma_{PTSN}$
**then** 16:      add $b_{best}$ to $\tau_{i}$; remove $b_{best}$ from $B^{t}$ and move $\tau_{i}$ to $C_{active}$ 17:      predict $b_{p}$ from $\tau_{i}$ and add $b_{p}$ to $B^{t+1}$ 18:    **else** 19:      **if**
$time_{waiting}\left(\tau_{i}\right)\geq \delta_{waiting}$
**then** 20:        move $\tau_{i}$ to $C_{die}$ 21:      **end if** 22:     **end if** 23:     **for**
$b_{j}\in B^{t}$
**do**
 24:      start a new tracklet with $b_{j}$ and insert it into $C_{lost}$ 25:     **end for** 26:   **end for** 27:  **end for** 28:  **for**
$\tau_{i}\in C_{active}$
**do** 29:     **if**
$len\left(\tau_{i}\right)\;\geq \lambda_{min}$
**then** 30:    add $\tau_{i}$ to $C_{final}$ 31:     **end if**32:  **end for**

## 4. Experiment

To evaluate the proposed method, we conducted extensive experiments on the VolleyTrack dataset. The database, implementation details, evaluation index, and results are described as follows.

### 4.1. Databases

The public benchmarks for MAT in sports videos are very limited compared to that for general MOT. In this study, we used the databases in [28], i.e., APIDIS, NCAA and VolleyTrack, which are newly collected and improved ones.

**Figure 6:**
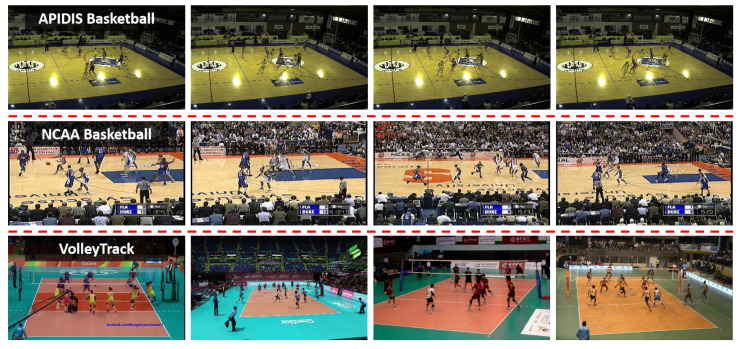
Example frames of the APIDIS, NCAA basketball and VolleyTrack databases (each row belongs to a database).

The APIDIS dataset contains 13 sequences of basketball games, each of which belongs to a round and lasts 10–20 s. It is collected from the original 15-min video on camera-6 (side view) (APIDIS, http://www.apidis.org/_Dataset/). As illustrated in Figure 6 (top row), the frames in APIDIS have difficult illumination conditions in background. The dataset totally contains 5764 frames of a resolution of 1600 × 1200 recorded at 22 fps. At each frame, seven persons (two referees and two five-player teams) on the court are annotated. In the experiments, seven sequences were used for training and the remaining ones for testing.

The NCAA Basketball dataset consists of NCAA basketball games from Youtube videos and is used for team activity recognition originally. Figure 6 (middle row) shows some example frames. To evaluate MAT methods, Kong et al. [28] manually annotate parts of data with bounding boxes of each player. Concretely, it contains four rounds in a game video, each of which lasts about 300 frames, at 30 fps. The sequences totally have 1179 frames, of a resolution of 640 × 480. Following [28], due to the small scale, it is not enough to train deep networks. Considering the similarity with APIDIS, we combined the APIDIS training set and two NCAA Basketball sequences for training, and the remaining two sequences were used for testing.

The VolleyTrack dataset contains 18 video sequences of world-class volleyball games collected from YouTube, as shown in Figure 6 (bottom row). Each video is captured by a camera equipped at the end line of the competition terrain, and there exist variations in background and illumination. The video sequences, corresponding to game rounds, last from 8 to 12 s. The dataset contains 5406 frames, at 30 fps, of a resolution of 1920 × 1080. The bounding boxes of players are manually annotated at each frame. Due to the same setting at both sides of the ground, the dataset only considers the athletes in the half ground near to the camera for each video. Among the 18 sequences, 50% of the data were for training and the others for testing.

We used the Faster R-CNN detector trained on each dataset to produce detections for all the evaluations.

### 4.2. Implementation Details

In our experiments, we set *H* (size of input vectors to LSTM) as 32 for all the three streams, but the source of the input vector was different. 64-dimensional input vector of PAS $\phi_{t}^{pa}$ consisted of 32-dimensional $\phi_{t}^{p}$ from the pose detector and 32-dimensional $\phi_{t}^{pa}$ from the ResNet; 32-dimensional input vector of PMS $\phi_{t}^{pm}$ came from the result processed by motion extractor; 64-dimensional input vector of PIS $\phi_{t}^{pi}$ was from expanding 8×8 Interaction Grid by column. The network hyper-parameters were chosen by cross validation and our framework is trained with Adam optimizer. The size of the LSTM hidden layer vector was 128. We trained our PTSN with a mini-batch of 64, and initially set the learning rate as 0.002 and decreased it by a factor of 0.1 in every 10 epochs. The PTSN was trained for 50 epochs.

As we know, it is hard to train a deep model on imbalanced datasets. Many works [38,39] deal with class imbalance issue, and achieves much progresses. In our work, we train PTSN as a 0/1 classification problem, where the negative pair belongs to class 0 and positive pair belongs to class 1. We used a resampling strategy to evade the imbalance classes. Specifically, for each pair, there existed one positive sample and many negative ones. Retain one of detections in the positive pair, the negative examples were constructed by replacing the other detection with another random athlete. We ran our model on a machine equipped with two Intel (R) Xeon E5-2620 v2 CPUs (12-core, 2.6 GHz), 16 GB RAM, and a 1080Ti GPU.

### 4.3. Evaluation Indexes

To evaluate the performance of multiple athletes tracking algorithms, we used metrics widely used in MOT [25]. Among them, Multiple Object Tracking Accuracy (MOTA) and Multiple Object Tracking Precision (MOTP) were two popular ones. According to [40], MOTA gives a very intuitive measure of the tracker’s performance at detecting objects and keeping their trajectories. MOTP shows the ability of a tracker to estimate precise object positions. In addition, there were some indicators that we used to measure the quality of the method. Mostly Tracked targets (MT) can be defined as the ratio of ground-truth trajectories that are covered by track predictions for at least 80% of their respective life span; Mostly Lost targets (ML) can be defined as the ratio of ground-truth trajectories that are covered by a track hypothesis for at most 20% of their respective life span; FP can be defined as the total number of false positive and FN can be defined as the total number of false negatives (missed targets). IDS is defined as the total number of identity switches [41].

### 4.4. Results Analysis

We first explored the contributions of different components in PTSN on the VolleyTrack dataset, as shown in Table 1. It can be seen that combining all three streams led to higher performance compared to using parts of them, indicating the effectiveness of pose-based appearance, motion and interaction clues. Compared to the counterpart that only uses appearance (AS), incorporating pose information, i.e., Pose-based Appearance Stream (PAS), gained about 8% in terms of MOTA, demonstrating the key role of pose cue. It can be seen that the one with motion stream (PMS) achieved better performance than with PAS and AS in terms of many metrics. Besides, the interaction clues had better ability in reducing ID switches, evidenced by PIS vs. PMS and PAS.

In Table 2, Table 3 and Table 4, we compared our method with the ones of other state-of-the-art MOT methods on APIDIS, NCAA Baseketball and VolleyTrack datasets respectively. It can be observed that the proposed approach achieved better scores than the other ones, including MHT_DAM [9], CEM [42], ELP [20], Siamese CNN [26] and MDPNN16 [6], on some metrics such as the IDF1 and FP. It indicates the effectiveness of the PTSN and multi-state matching algorithm for multi-athlete tracking in sports videos. Note that some method, e.g., CEM [42], ELP [20] and Siamese CNN [26], woredk in offline association mode, and made use of much more context information. Our method could obtain comparable results with those offline association methods. More importantly, thanks to the online matching, our method had the superiority in processing speed (higher FPS).

**Figure 7:**
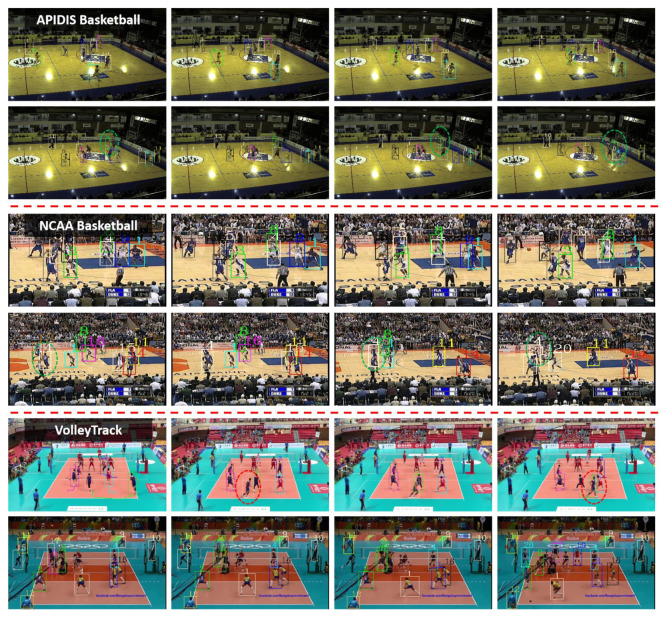
Visualization tracking results on APIDIS, NCAA Basketball and VolleyTrack databases (each row belongs to a sequence). The athletes in green dashed circles are success cases and the ones in red dashed circles are failure cases.

Figure 7 illustrates some qualitative results, containing both success and failure examples. By using long-term dependencies of pose-based clues, our method could largely recover the target after an occlusion. When the detections were missing due to occlusion or approaching of athletes, our method could track the ones with the same identity, e.g., the athletes in green dashed circles. Meanwhile, we can also notice that our method tended to fail in some difficult situations, e.g., the athletes in red dashed circles dressed very similarly.

## 5. Conclusions

In this paper, we propose a novel online multiple athlete tracking approach in sports videos. It makes use of long-term temporal pose dynamics for better associate correct athlete targets. First, we propose the Pose-based Triple Stream Networks, which models the pose dynamics by three clues, i.e., pose-based appearance, motion and interactions among athletes. Second, we design a multi-state online matching algorithm based on bipartite graph matching. Due to the multiple detection states and reliable transitions, it is robust to noisy detections and occlusions. The proposed method is evaluated on the APIDIS, NCAA basketball and VolleyTrack datasets by comparing to other popular MOT methods, and the experiment results clearly demonstrate its advantages for this task. The proposed PTSN and online matching algorithm are separate modules, which limit the practicability in MAT/MOT. In the future, we aim at building an integrated pipeline in which both feature extraction and data association can be jointly learned.

## Figures and Tables

**Table 1:** Comparison of different components of Pose-based Triple Stream Network (PTSN) on the VolleyTrack.

Components	MOTA↑	MOTP↑	MT↑	ML↓	FP↓	FN↓	IDS↓
AS	71.3	62.5	41.67	49.12	954	2191	578
PAS	79.5	68.2	43.77	40.11	502	1488	394
PMS	80.5	70.1	45.40	41.30	450	1100	278
PIS	77.3	69.1	43.00	45.70	498	1510	147
PAS + PMS	80.9	71.0	50.34	35.09	438	1086	153
PAS + PMS + PIS	84.7	76.4	56.60	33.30	296	792	54

**Table 2:** Comparison with state of the art on the APIDIS dataset.

Methods	Mode	IDF1↑	MOTA↑	MOTP↑	MT↑	ML↓	FP↓	FN↓	IDS↓	FPS ↑
MDPNN16 [6]	Online	53.6	74.1	80.1	55.6	21.5	768	2812	192	1.2
CEM [19]	Offline	47.0	64.2	77.1	45.6	22.8	1506	3037	185	1.1
MHT_DAM [9]	Offline	49.3	73.5	79.1	50.7	23.2	863	2785	231	0.8
ELP [20]	Offline	57.0	76.0	80.8	56.6	21.0	794	2559	197	3.7
Siamese CNN [26]	Offline	54.4	75.6	80.7	56.3	22.2	716	2664	213	6.2
Ours	Online	58.0	75.2	80.5	52.6	21.0	748	2967	237	30

**Table 3:** Comparison with state of the art on the NCAA Basketball sequences.

Methods	Mode	IDF1↑	MOTA↑	MOTP↑	MT↑	ML↓	FP↓	FN↓	IDS↓	FPS ↑
MDPNN16 [6]	Online	42.2	73.2	74.9	45.0	5.0	174	1133	85	1.7
CEM [19]	Offline	36.1	50.8	52.1	25.0	15.0	831	1698	70	1.5
MHT_DAM [9]	Offline	44.5	69.2	68.6	35.0	10.0	153	1140	84	1.1
ELP [20]	Offline	44.8	75.8	77.4	45.0	5.0	167	1008	86	4.3
Siamese CNN [26]	Offline	44.4	75.2	76.9	45.0	0	164	1033	91	7.6
Ours	Online	48.5	72.2	73.6	35.0	5.0	133	1240	74	34

**Table 4:** Comparison with state of the art on the VolleyTrack dataset.

Methods	Mode	IDF1↑	MOTA↑	MOTP↑	MT↑	ML↓	FP↓	FN↓	IDS↓	FPS ↑
MDPNN16 [6]	Online	78.3	72.7	64.0	45.5	18.3	560	882	85	1.1
CEM [19]	Offline	82.8	80.1	76.2	57.1	11.4	378	726	68	1.1
MHT_DAM [9]	Offline	80.9	84.9	76.3	55.3	35.1	314	818	94	0.7
ELP [20]	Offline	84.4	83.3	75.1	54.3	28.2	325	748	63	2.6
Siamese CNN [26]	Offline	81.4	83.3	75.2	55.7	18.2	375	768	93	6.0
Ours	Online	85.0	84.7	76.4	56.6	33.3	296	792	54	28

## Data Availability

Not applicable.
